# Impacts of the Russia-Ukraine War on Global Food Security: Towards More Sustainable and Resilient Food Systems?

**DOI:** 10.3390/foods11152301

**Published:** 2022-08-02

**Authors:** Tarek Ben Hassen, Hamid El Bilali

**Affiliations:** 1 Program of Policy, Planning, and Development, Department of International Affairs, College of Arts and Sciences, Qatar University, Doha 2713, Qatar; 2 International Centre for Advanced Mediterranean Agronomic Studies (CIHEAM-Bari), Via Ceglie 9, Valenzano, 70010 Bari, Italy; elbilali@iamb.it

**Keywords:** war, conflict, Ukraine, Russia, food security, export restrictions, food supply, SDG

## Abstract

As a conflict between two major agricultural powers, the Russia–Ukraine war has various negative socioeconomic impacts that are now being felt internationally and might worsen, notably, for global food security. If the war deepens, the food crisis will worsen, posing a challenge to many countries, especially those that rely on food imports, such as those in the Middle East and North Africa (MENA) region. Simultaneously, the war came at a bad time for global food markets because food prices were already high due to disruptions in the supply chain caused by the COVID-19 pandemic, strong global demand, and poor harvests in some countries. Understanding how conflict-related disruptions in global food and fertilizer markets might affect price and availability is critical for understanding the overall impact on global food security. Further, four months into the war, its implications for food security suggest that this review is timely, urgent, and highly needed. Accordingly, this paper aims to investigate the Russia–Ukraine war’s direct and indirect impact on global food security. The paper highlights that the war resulted in immediate and far-reaching cascading consequences on global food security: Ukrainian exports have stopped, conscription and population displacement have caused labor shortages, access to fertilizers is restricted, and future harvests are uncertain. First, Ukraine’s export capacity has been hampered. Secondly, conscription and population displacement caused labor shortages. Thirdly, access to vital agricultural products such as fertilizers is also constrained. The war may delay spring planting and winter crop harvesting. Further, the war has indirect and cascading effects. Indeed, rising fertilizer costs may reduce their use and crop yields. Moreover, as seen during the 2007–2008 food crisis, export restrictions and speculation are driving up international prices and worsening the situation. Furthermore, the war triggered a panic buying movement at country and individual levels. Finally, the war may jeopardize the implementation of the Sustainable Development Goals (SDGs), notably SDG 1 (No poverty), SDG 2 (Zero hunger), and DG 12 (Responsible consumption and production). However, the consequences of the war on food security are being exacerbated by a variety of underlying rigidities, vulnerabilities, and inefficiencies in global food systems. Accordingly, the transition toward healthy, equitable, and ecologically sustainable food systems must be strengthened by adopting urgent and long-term reforms and policies.

## 1. Introduction

Food security happens when “all people at all times have physical and economic access to sufficient, safe, and nutritious food to meet their dietary needs and food preferences for an active and healthy life” [1]. Food security has four standard dimensions: availability (having a sufficient quantity of food available regularly); access (having enough resources to acquire suitable and healthy food); utilization (having a reasonable food use based on knowledge of essential nutrition and care); and stability of availability, access, and utilization of food [2,3]. Although these four dimensions remain fundamental, they lack other features, such as agency and sustainability, that have come to be recognized as critical for altering food systems in the direction required to accomplish the SDGs [4]. Evidence shows that wars and conflicts are the most important drivers of food insecurity globally [5]. Indeed, in 2021, 139 million people were in crisis or severe food insecurity in 24 countries and territories, with war and instability being the primary drivers [6].

In the early hours of 24 February 2022, Russia started a full-scale military invasion of Ukraine that resulted in the deaths and injuries of civilians, as well as the destruction of key infrastructure [7]. Consequently, the United States, Europe, and many other western countries (e.g., Canada and Australia) have imposed increasingly broad sanctions, targeting persons, banks, corporations, and large state-owned companies, as well as exports [8]. The most significant consequences of the war are the lives lost and the humanitarian disaster in Ukraine caused by many besieged and displaced people. Simultaneously, the war has inflicted a significant blow to commodities markets, especially food and energy, affecting global patterns of trade, production, and consumption in ways that will maintain prices at historically high levels until the end of 2024, thus threatening global food security [9,10,11].

Indeed, in the context of globalized agricultural markets and as a war between two major players in the global food and fertilizer industries, the war is raising widespread anxiety about global food security [12,13]. Despite their limited position in the global economy, with only approximately 2% of global GDP, Russia and Ukraine are considered both ‘global breadbaskets’ and are important producers and exporters of vital agricultural commodities, minerals, fertilizers, and energy, where exportable resources are often concentrated in a few countries [9]. This concentration may make these markets more vulnerable to shocks and volatility [14]. Simultaneously, the war came at a bad time for global food markets because food prices were already high due to disruptions in the supply chain caused by the COVID-19 pandemic, strong global demand, drought, and poor harvests in South America the previous year. These factors combined drive up food prices [15]. These issues will worsen due to the war between Russia and Ukraine.

Indeed, four months into the war, the consequences are clear: Ukrainian exports have ceased, future harvests are questionable, and global agricultural commodity prices have skyrocketed, threatening to push millions into hunger and poverty [13]. Further, price increases and trade interruptions might increase the number of malnourished individuals by limiting the availability of humanitarian assistance to prevent and cure acute malnutrition [16]. The World Food Programme (WFP) estimates that acute hunger will grow by an additional 47 million people from a pre-war baseline of 276 million people suffering from acute hunger. This indicates that up to 323 million people may face severe food insecurity by 2022 [6,17]. According to World Bank estimates, every one percentage point rise in food prices pushes 10 million people into severe poverty. If food costs remain this high for a year, global poverty might rise by more than 100 million [18].

There is a considerable amount of uncertainty regarding the effect of the war on food security in the medium (6 months–2 years) to long term (>2 years). This includes both the immediate costs of the war and the implications of Russia’s current and future sanctions [12]. In that context, the combined impacts of sanctions and war will have a wide-ranging impact on global agri-food markets and food security, sending shockwaves worldwide, especially in import-dependent low- and middle-income countries (LMICs) [19]. For instance, several countries in the Middle East and North Africa (MENA) region import more than 50% of their cereal needs, especially wheat, from Ukraine and Russia [19].

Understanding how conflict-related disruptions in global food and fertilizer markets might have large-scale and long-term implications on price and availability is critical for understanding the overall impact on global food security. Further, the effects of the war on food systems and supply chains worldwide suggest that this review is timely, urgent, and highly needed. However, evaluating the consequences of the war on food security is challenging since the entire extent of the war’s effects is not yet clear. Accordingly, this paper aims to investigate the Russia–Ukraine war’s direct and indirect impacts on global food security.

The paper is based on a review of grey literature, including reports, policy documents/briefs, and working/discussion papers produced in English, French, and Arabic by a variety of organizations, including international organizations (e.g., FAO, World Bank, World Food Programme (WFP), International Food Policy Research Institute (IFPRI), International Monetary Fund (IMF), United Nations Conference on Trade and Development (UNCTAD), United Nations Development Programme (UNDP), Organisation for Economic Co-operation and Development (OECD)), regional organizations (e.g., United Nations Economic and Social Commission for West Asia—ESCWA, etc.), NGOs (e.g., Oxfam), consulting firms (e.g., Deloitte, KPMG, McKinsey, Oxford Business Group), international newspapers (e.g., *The New York Times*, *Le Monde*, *Malay Mail*, *The Guardian*, and *The Independent*), and international news platforms (e.g., *Bloomberg*, and *Euronews*).

## 2. Overview of the War’s Immediate and Long-Term Impacts

**Figure 1:**
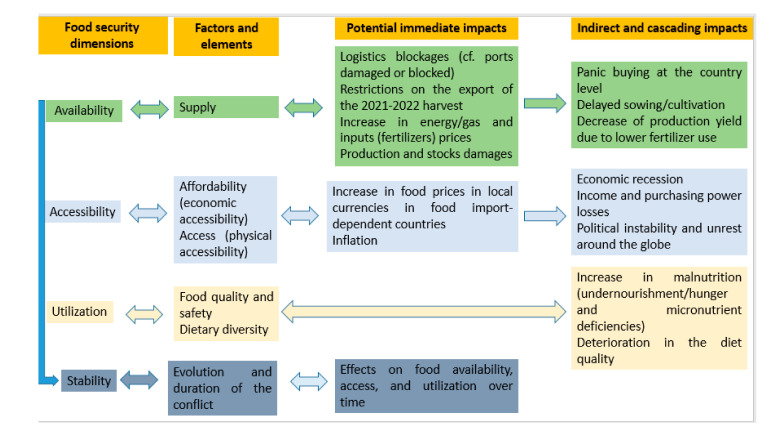
Impacts of the Russia–Ukraine war on global food security. Source: Authors’ elaboration.

The war has a multitude of immediate and long-term indirect impacts on global food security (Figure 1).

### 2.1. Immediate Impacts of the War on Food Security

The war has a multitude of direct and immediate consequences for food security, disturbing harvesting and shipping and severely affecting staple supplies and pricing [20]. Firstly, military actions might have both short- and long-term consequences on Ukraine’s ability to transport agricultural products inside and beyond its borders, especially if port facilities and railroads are destroyed. In fact, the war immediately affected grain shipments from Ukraine, mainly for maize, typically in the spring and early summer. Indeed, 95% of Ukrainian grain exports are sent by sea via the ports of Odessa, Mariupol, and Kherson, which have suffered significant damage. In addition, all Black Sea ports have been blocked, shutting off most Ukrainian exports. Shipping grain by rail would be complicated even if inland transportation infrastructure remained intact due to a lack of an operable railway system. For instance, according to Reuters [21], on 17 May 2022, four traders announced that about 300,000 tonnes of Ukrainian wheat contracted by Egypt’s state grains buyer for delivery in February and March were stranded in Ukraine, with one cargo detained in port and four others needing to be loaded [21].

Instead of utilizing Ukrainian ports, potential options include exporting food through Poland or Romania. In recent weeks, Western leaders have queued up to endorse these options. Alternative methods may enhance exports, but experts think this is insufficient to fulfill global food demand. Indeed, challenges are numerous: the rail gauge in Ukraine differs from that of most EU nations. Building storage capacity will take some time. Further, the Romanian port of Constanta lacks the capacity to manage the influx of Ukrainian crops [22]. However, with investments in port infrastructure and railway networks, it can be a key trade route, especially to North African countries. In addition, because the embargo duration is unknown, attracting private investment for the infrastructure required for such alternatives is challenging [22]. Furthermore, increased insurance costs for the Black Sea area will worsen already high transportation expenses, compounding the price of food imports [23].

Secondly, the war already prevented farmers from working in their fields, and the conscription and population displacement resulted in labor shortages. Disruptions to essential public services are also expected to affect agricultural activities negatively. This situation is aggravated by reduced access to and availability of critical agricultural inputs, such as fertilizers [24]. Accordingly, the war might disrupt the ongoing spring planting campaign and the approaching winter crop harvesting, which usually takes place in June/July [24]. For instance, although the available quantities of seeds (both local and imported) would be adequate to plant 70% of the predicted spring area, their safe delivery to farmers is a considerable challenge [25]. Consequently, according to FAO [19], one-third of crops and agricultural land may not be harvested or cultivated by 2022. Further, it is uncertain if other exporters will be able to fill in the gap.

Thirdly, due to economic sanctions placed on Russia, there is a great deal of uncertainty about Russian export prospects in the future [14]. Russian Black Sea ports remain open for the time being, and no significant interruption to agricultural production is predicted in the near future. However, the financial sanctions imposed on Russia have resulted in a significant devaluation, which, if sustained, may impede productivity and development while eventually raising agricultural output costs [23]. Furthermore, in April 2022, Russia vowed to limit agricultural and food exports to only ‘friendly’ countries in response to Western sanctions. The restriction would exacerbate the global food-supply shortfall [26]. A continuing war and sanctions would probably raise prices and weaken food security for hundreds of millions of people [15].

### 2.2. Indirect Impacts of the War on Food Security

The war has as well some indirect and cascading consequences. Firstly, prices for essential inputs, such as fertilizers, are reaching near-record highs [27]. Consequently, many farmers worldwide, such as in the USA, are replacing high-cost fertilizer-requiring crops, such as wheat and maize, with low fertilizer-requiring ones, such as soy. Since soybeans are used mainly in animal feed and biofuel, this might exacerbate the current supply shortages and raise the price of bread, cereals, and other critical food items [28]. Similarly, fertilizer shortages and high costs may have negative effects, particularly in developing countries where price repercussions may severely limit usage, and result in decreased yields during reduced global supply and record global prices [29].

Secondly, as seen during the 2007–2008 food crisis, many countries applied export restrictions to secure local food supply and mitigate inflation (India: wheat; Serbia: grains, and vegetable oils; Indonesia: palm oil, etc.), forcing other food exporters to limit exports to protect their populations as well, exacerbating the situation [30]. Since the beginning of the war, the number of countries enforcing food export restrictions, such as export bans and export licensing requirements, has increased from 3 to 26, covering 40 food items [31]. The entire volume of exports impacted by the restrictions accounts for around 15.68% of total calories traded globally, the same level seen during the 2007–2008 food crisis [31]. Export limits are especially significant in terms of calories for the following commodities: wheat (31% of total calories impacted), palm oil (29%), maize (12%), sunflower oil (11%), and soybean oil (6%). In terms of total trade in individual items, export restrictions have an impact on 36% of wheat exports, 55% of palm oil exports, 17% of maize exports, 78% of sunflower oil exports, and 6% of soybean oil exports [32]. However, while these measures may appeal locally, they have far-reaching implications for global food pricing and food security [30].

As seen during the 2007–2008 food crisis, instead of controlling price rises, export restrictions pushed up international market prices [33]. Indeed, rising protectionism is compounding the instability in global food markets caused by the war. These measures can potentially have disastrous unintended repercussions for vulnerable individuals in food-importing nations, raising prices and intensifying food insecurity concerns already exacerbated by the COVID-19 outbreak [31]. Export restrictions exacerbated shortages during the 2007–2008 food crisis, leading to food riots across Asia and Africa [34].

Thirdly, panic buying at both country and individual levels is another cascading impact of the war. Stockpiling and panic buying are significant components of crisis- and disaster-related consumer behavior that attracted substantial media attention during the COVID-19 pandemic [35]. Indeed, food becomes more important in people’s lives during a crisis, and panic buying is a natural human reaction to a stressful scenario. As observed during the first months of the pandemic [36,37,38,39], in March 2022, several European countries saw a rise in panic buying due to the war. For example, in the United Kingdom, more than a third of the consumers hurried to stock up on critical products, such as pasta and cooking oils, while numerous retailers started to ration certain food items [40]. Likewise, residents in northern Italy stored pasta in large quantities, while trade experts in Germany reported panic buying of commodities [41]. Further, residents in Finland’s border areas were hurrying to buy food in preparation for a possible war with Russia [42]. Furthermore, to ensure local food supply, some countries were stocking up food, such as China, a lesser-known driver of food prices increase. The Chinese government is hoarding food on a vast scale in order to avert shortages and minimize reliance on imports. According to the US Department of Agriculture (USDA) data, by mid-2022, China, which accounts for less than 20% of the world’s population, is expected to have 69% of the globe’s maize reserves, 60% of its rice, and 51% of its wheat. The forecasts imply rises of roughly 20% over the last decade, and the data plainly reveals that China continues to store grains, contributing to higher global food prices [43].

Fourthly, by slowing the post-COVID-19 economic recovery, the war may affect purchasing power at the country and individual level, affecting economic access to food. In fact, the war in Ukraine erupted at the worst possible time for the global economy, which was still reeling from the effects of the COVID-19 pandemic. The war exacerbated the dire global economic and social circumstances [44]. Before the war, post-pandemic recovery was predicted to continue in 2022 and 2023, backed by sustained global vaccine efforts, supportive macroeconomic policies, and favorable financial conditions, despite high inflation in several countries [9]. However, the war created a new negative shock for the world economy, affecting the global market of food, energy, and other commodities, fueling ongoing inflation, and prompting a global food crisis. In April 2022, the International Monetary Fund [45] predicted that global growth would fall from an expected 6.1% to 3.6% in 2022 and 2023. Additionally, food and fuel prices would increase by 3% in 2022 and 2.3% in 2023. This might have significant societal ramifications since rising food and energy costs will have a disproportionate impact on the poor and middle classes [20].

Fifthly, the rise in international prices has placed pressure on food-importing nations’ foreign reserves and, as a result, their exchange rates. Most food import-dependent nations are already heavily indebted; before the crisis, developing countries spent an average of 16% of export profits on debt service [46]. For instance, as of April 2022, against the dollar, the Egyptian pound had depreciated by 17%, the Moroccan dirham by 4.5%, the Tunisian dinar by 3%, and the Lebanese pound by 25%. Currency depreciation is anticipated to negatively influence inflationary pressures on food and other commodities and services, reducing the buying power of consumers’ earnings and adding to the load on governmental budgets. In February–March 2022, food costs had already risen in various countries. In Egypt, for example, food costs increased by an estimated 17% in February 2022 [20].

Additionally, many importing countries are more vulnerable than others and depend on Ukraine and Russia’s food supplies. For instance, the Middle East and North Africa (MENA) region countries import more than 50% of their cereal needs and a large part of the wheat, maize, and barley from Ukraine and Russia. Lebanon, for example, obtains 80% of its wheat from Ukraine [19]. This might lead to increased food insecurity and poverty in countries where diets are dominated by government-subsidized bread, such as Egypt and Lebanon [47]. The war in Ukraine also reduces food supplies in the region at a time when governments have little budgetary flexibility to buffer the impact of increased food costs due to economic constraints caused by COVID-19 restrictions [10]. In many low- and middle-income countries (LMICs), governments’ capacities to maintain their social safety-net programs and continually subsidize essential food products will be tested [48]. In addition, low-income families will be particularly affected by increased food prices since they spend most of their income on food [49]. For instance, in Egypt, the government spends around $3 billion a year on wheat imports, most of which goes toward a bread subsidy program, the Tamween ration card system, covering 73% of Egyptian families [50]. The recent spike in wheat prices has resulted in a significant increase in the expense of administering this program [49].

Furthermore, commodity speculation is another factor exacerbating these price shocks. However, ‘extreme speculation’ might cause bigger upward movements than would have been the case based only on supply-and-demand factors. For food, this means higher real-world prices that harm the world’s poorest people, but for other commodities this means larger profits or losses for investors. In 2007–2008, a significant surge of speculative financial investment led to skyrocketing futures prices [46]. A Lighthouse Reports study discovered a massive flood of investor capital into specialized agricultural funds, most of it coming from speculators who have nothing to do with the physical production or distribution of wheat but saw a chance to make fast profits. According to the ‘Hunger Profiteers’ investigation, increased speculation in the commodities markets by investment corporations and funds has led to the price increase. For instance, by 11 April 2022, the top two US agricultural funds, Invesco’s agriculture fund and Teucrium’s wheat fund, had received a net investment of $1.2 billion, compared to $197 million for the whole year of 2021. By mid-April, they had contracts for wheat futures worth over half of the UK’s annual flour consumption [51].

Finally, the war will delay many countries’ sustainable transformation of food systems. Several countries are pushing Europe to postpone the transition to greener agriculture to increase agricultural output in response to the war. Indeed, the European Commission said in March 2022 that the publication of recommendations on sustainable farming and the environment would be delayed. With the effect of the Ukrainian war on food supplies, several nations are questioning the European Union’s environmental effort [52]. Further, the EU’s “Farm to Fork” policy, which seeks to cut pesticide usage in half, reduce fertilizer use by 20%, and commit a quarter of agricultural land to organic farming by the end of the decade, was set to be published in legislative texts in March 2022. It has been postponed indefinitely [53]. Due to high prices, in Brazil, some federal politicians are attempting to open protected indigenous territories for potash mining [54].

As a matter of fact, and as observed during the pandemic [55], the war may have an impact on the progress toward the UN Sustainable Development Goals (SDGs). Indeed, by creating a global food crisis, the war may jeopardize the SDGs’ implementation, including SDG 1 (No Poverty) and SDG 2 (Zero hunger). Additionally, high energy costs have driven numerous governments to boost fossil fuel production, thus delaying the shift to renewable energy. For instance, fossil fuels are experiencing a wartime revival, with governments more concerned with lowering the price of oil and gas than with decreasing emissions quickly. In addition, governments are abandoning efforts to phase out coal use. They are scurrying for additional oil and devoting billions of dollars to the construction of liquefied natural gas facilities [56]. Furthermore, rising metal prices are increasing renewable energy costs, which depend on metals such as aluminum and battery-grade nickel [11]. This may jeopardize the implementation of SDG 12 (Responsible sustainable consumption and production).

## 3. The Impact of the Ukraine Crisis on the Global Agri-Food Markets

**Figure 2:**
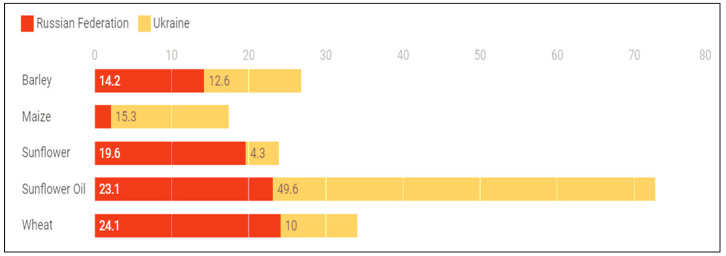
Shares of Russia and Ukraine in the global trade of selected commodities (2018–2020). Source: Glauber and Laborde [27].

Following the dissolution of the Soviet Union in the early 1990s, agricultural productivity and output declined, and Russia and Ukraine became net food importers [57]. However, after intensive modernization and mechanization during the last three decades, Russian and Ukrainian agriculture outputs and food commodities exports have increased significantly, making the region the world’s breadbasket. The two countries are now among the world’s top producers of various agricultural products, mainly cereals and sunflower oil. Together, in 2020, they account for 72.7% of global trade in sunflower oil and seeds and 34.1% of global trade in wheat [58]. About 12% of the world’s total caloric commerce is exported by Russia and Ukraine [27] (Figure 2).

### 3.1. Impact of the War on the Global Cereals Market

**Figure 3:**
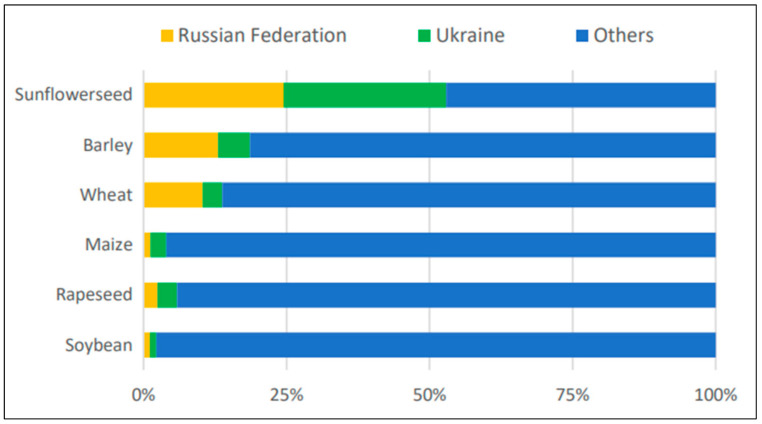
Share of Russia and Ukraine in global exports of selected crops. Source: FAO [19].

Regarding cereals, the contribution of Russia and Ukraine to the global supply is notably substantial for barley, wheat, and maize. Between 2016/17 and 2020/21, the two nations accounted for 19%, 14%, and 4% of the global production of these crops, respectively. In 2021, Russia and Ukraine were among the top three global wheat and maize exporters [14] (Figure 3).

Likewise, due to economic sanctions placed on Russia, there is a great deal of uncertainty about Russian export prospects in the future [14]. The early production outlook for 2022/23 winter crops is favorable in both countries. However, as explained above, the war may harm agricultural operations in Ukraine, prohibiting farmers from attending to their fields, and harvesting and selling their products [14].

Wheat occupies a central place in human nutrition. It is an essential food for more than 35% of the world’s population, providing 20% of the daily protein and food calories. In North Africa and West Asia, wheat provides about 40–43% of the daily calories and proteins [59]. The present war may result in a sharp drop in wheat exports from Russia and Ukraine. The great uncertainty concerns the next campaign, which starts in July 2022. If shipments do not restart quickly, the silos will not be available to reap the summer harvests. The most dramatic situation would be when the continuation of the war would hamper the next Ukrainian harvest. Global stocks will not fill such supply disruptions for long. Moreover, there is hardly any untapped production potential globally in the short term. It is uncertain if other exporters can fill in the gap. Wheat supplies in Canada are already limited, and exports from the United States, Argentina, and other nations are likely constrained as the government attempts to assure local supply [19].

The impacts on the maize market depend mainly on Ukraine since Russia is a small producer. Ukraine is the world’s fourth-largest maize exporter, accounting for around 15% of the global market. Following the droughts that had an impact on output in Brazil and Argentina, the market is tight, and the war jeopardizes the next crop and Ukraine’s ability to export [15]. The first question for maize is about sowing in the spring of 2022 (April–May) for an autumn harvest. If the war prevents sowing, the world corn market, already very tight, will not be able to compensate for the lack of deliveries from Ukraine, with, in perspective, an additional increase in the cost of animal feed and difficulties for the food supply in Latin America. Furthermore, following supply concerns in maize and wheat, the war has raised feed demand for rice, driving rice prices into areas of extreme volatility. In the Asian rice market, domestic and international feed demand inflow has curtailed broken rice supplies [60].

Before the war, global food prices had already hit an all-time high. This was primarily due to market circumstances, but it was also owing to expensive energy, fertilizer, and other agricultural service costs. In February 2022, the FAO Food Price Index (FFPI) (the FAO Food Price Index (FFPI) measures the monthly change in international food commodity prices—it is made up of the average of five commodity price indices (viz. cereal, vegetable oil, dairy, meat, and sugar), weighted by the average export proportion of each category from 2014 to 2016) reached a new record high, 21% more than the previous year and 2.2% higher than the previous high in February 2011. In March 2022, the FFPI averaged 170.1 points, up 24.9 points (17.1%) compared to February 2022, marking its highest level on record since 1990 [19]. Since the beginning of the war, wheat and maize prices have risen by 35%, while overall food prices have increased by 5% globally [20]. On average, in April 2022, the FFPI fell by 1.2 points (0.8%) from its all-time high in March 2022 but was still 29.8% higher than its value in April 2021. While grain prices fell somewhat, vegetable oil prices took the biggest hit in April’s Food and Feed Price Index (FFPI). Sugar, meat, and dairy products all saw modest rises in their pricing sub-indices [61].

The consequent supply shortages for importers may be particularly relevant for purchasers in the Near East and North Africa, and, given the significance of wheat as a dietary staple, some nations may increase imports now to secure supplies out of worry that wheat markets will tighten and prices will rise higher. This would add to the strain on global markets [19]. With agricultural prices on the rise, governments across the globe are attempting to secure local cereals supply. For instance, India suspended wheat exports on 14 May 2022, with immediate effects to ensure its national food security and domestic availability and manage inflation. Following the war in Ukraine, India sought to cover the global wheat supply shortfall and intends to export a record 10 million tonnes in 2022–2023 [62]. However, a record-breaking heatwave during India’s warmest March on record has reduced this year’s wheat harvest, potentially cutting production by up to 50% in some regions of the country [63]. India’s export ban has been denounced by the G7 developed countries, who believe it would exacerbate the crisis [64]. Indeed, the impact of India’s decision was immediate: prices on the European market skyrocketed on Monday, 16 May 2022. At the closure, the wheat price hit 438 euros/tonne [65]. Likewise, in April 2022, the Serbian government announced a limitation on the quantities of wheat, maize, flour, and cooking oil planned for export to mitigate the risks of market disruptions caused by rising demand on both the international and domestic markets [66].

### 3.2. Impacts of the War on the Global Vegetable Oil Market

Palm oil (58%), soybean oil (14%), sunflower oil (13%), and rapeseed (canola) oil (7%) together account for 92% of vegetable oils sold in global markets on average between 2019 and 2021 [67]. Due to several factors, such as protracted global supply tightness and robust demand, global vegetable oil supplies have tightened for the last two years, and, consequently, prices have been steadily rising. In 2021, the prices increased by 65% and 63% for rapeseed oil and sunflower oil, respectively [19]. Additionally, drought in South America has reduced soybean yields, notably in Brazil, a major producer. Malaysia’s palm oil output fell in December 2021 owing to Typhoon Rai, severe labor shortages, and other challenges exacerbated by COVID-19-related restrictions on worker migration. In Canada, the major exporter of rapeseed oil, drought-affected rapeseed output fell 35% in 2021/22. Therefore, Canada’s rapeseed exports are expected to drop by half and rapeseed oil exports by 20% [67].

The war prompted prices to rise in turbulent trade as the war disrupted Black Sea sunflower oil exports. Russia and Ukraine are significant exporters of sunflower oil, and the crisis has dramatically driven up vegetable oil prices. In the absence of other routes to the west through Romania or Poland, about half of last year’s harvest’s sunflower oil remained in Ukraine. Much of Ukraine’s sunflower agricultural region sits east of the Dnieper River, where much of the fighting has since ceased. Due to Black Sea marine trade restrictions, Russian export quotas, and sanctions on corporate operations, Russian exports are now restricted. Sunflower oil has been the most immediately affected, with a more than 40% rise since the invasion. It accounts for around 13% of all vegetable oils sold worldwide, with Ukraine and Russia accounting for over 50% and 25% of all sunflower oil marketed globally, respectively. Because vegetable oils do not need much processing, high costs have already been passed on to consumers and retailers [67].

Consequently, in several countries, such as the United Kingdom, some consumers are being limited in their cooking oil purchases as shops and restaurants respond to rising prices. Indeed, grocery chains in Spain, Greece, Turkey, Belgium, and other countries have restricted cooking oil purchases [68]. The issue has also sparked trade policy reactions throughout the globe, further restricting supply and raising costs [67]. For instance, end of April 2022, Indonesia bans palm oil exports amid domestic price rises, putting more upward pressure on global cooking oil prices. The ban will see Indonesia restrict exports of all cooking oils and related raw materials to decrease local shortages and reign in price increases that potentially cause significant domestic unrest [69]. Meanwhile, the world’s second-largest palm oil producer after Indonesia, Malaysia, is still suffering from a chronic labor shortage, having an impact on farmers’ yields and output [70].

### 3.3. Impact of the War on the Global Fertilizer Market

Despite the efforts to reduce nutrient losses to the environment, fertilizers are still a crucial part of agricultural productivity [29]. In general, farmers must use three main kinds of mineral fertilizers to guarantee crop growth: nitrogen (N), phosphate (P), and potash (K). All three of these mineral fertilizers are marketed on a global scale, and their supply is geographically concentrated and controlled by a small number of miners (P and K) and a somewhat larger group of chemical businesses (N) [71].

Russia and Belarus are significant potash mining and production countries, and Russia is a considerable nitrogen supplier. In 2020, Russia was the world’s top fertilizer exporter, with an estimated $7.6 billion in exports [72]. Around one-sixth of the world supply of potash fertilizers is exported by Russia, as is more than one-tenth of nitrogen fertilizer exports and around one-sixth of mixed fertilizer exports (containing two or more of nitrogen, potassium, and phosphate) [73]. In 2020, Belarus exported $2.96B in fertilizers, making it the sixth largest exporter globally. In the same year, Belarus was responsible for around 17.6% of the global potash production (K) [74]. Both Russia and Belarus are part of a cartelized potash market that accounts for one-third of worldwide exports and determines the price of potash on the global market (the other half consists of Canada and the USA) [73]. In addition, Russia is a large natural gas supplier, the primary raw material for nitrogen fertilizer. This is most significant to the EU and India, which rely heavily on imported natural gas for their domestic nitrogen production [71].

Before the war, the global fertilizer market was already under severe stress [75]. Surging energy costs, supply curtailments, and trade policies have pushed fertilizer prices up by 80% during 2021, reaching unseen levels since the 2008–2009 global financial crisis [11,76]. Firstly, fertilizer prices had been rising hand-in-hand with energy prices throughout 2021. As nitrogen-based fertilizers are produced from natural gas (or coal in the case of China), the price of natural gas soared in 2021, pushing some fertilizer prices to their highest level since 2010 [75]. For instance, ammonia production in Europe, an essential input for nitrogen fertilizers, was severely curtailed due to soaring natural gas costs [76]. Several fertilizer companies in Europe were already battling to continue their operations due to rising gas costs, with two UK factories closing in 2021 [77]. For instance, in March 2022, the fertilizer giant Yara announced reducing its European ammonia and urea production capacity by 55% due to rising gas prices [29].

Secondly, additional constraints on supply arising from trade policy measures taken by individual countries put more pressure on the global market. For example, China, a major producer and supplier of phosphate-based fertilizers, has decided to restrict fertilizer exports from July 2021 through June 2022 to ensure domestic supply [73]. Further, following several countries’ sanctions on Belarus in 2021, the global potash market faced more turbulence [76]. Later, following the war, on 8 April 2022, the European Union restricted importing fertilizers from Russia and Belarus as part of a larger package of economic sanctions [32].

The war in Ukraine has left the world not only short of important grains but also fertilizers, which could tighten food supplies. During the first quarter of 2022, the World Bank’s Fertilizer Price Index climbed over 10% (q/q) to an all-time high in nominal terms [11]. International benchmark prices of fertilizers rose similarly throughout 2021, with many quotations reaching all-time highs. The most notable increases were registered for nitrogen fertilizer. Prices of urea, an essential N fertilizer, have risen by two and a half times over the past 12 months, with prices of phosphorous fertilizer rising in tandem over the same period, while those of potash (K-fertilizer) remained less affected.

Much of South and Central America, West Africa, and Europe—including Ukraine— rely heavily on Russia and Belarus for their fertilizer imports, especially potash. In addition, Russia dominates in exporting natural gas to fuel the production of nitrogenous fertilizers across Europe. The International Energy Agency (IEA) reports that Gazprom’s gas exports to Europe decreased by roughly 25% in the last three months of 2021 compared to the same time in 2020 as tensions rose [73]. Roughly 20% of global trade in natural gas comes from Russia, while it contributes about 40% of EU imports. Natural gas prices might rise considerably more if sanctions halt trade. Additional fertilizer shortages may have global consequences, especially in developing countries where price repercussions might dramatically restrict usage and result in poor local harvests during lower global supplies and record global prices. Indeed, higher fertilizer prices make the world’s food supply more expensive and less abundant, as farmers skimp on nutrients for their crops and get lower yields [71]. For instance, given Africa’s still-limited fertilizer usage (an estimated average of 25 kg per hectare, a fraction of the worldwide average of 121 kg/ha), a decrease in fertilizer use would result in much lower production for the continent, possibly with significant ramifications for food security [29].

## 4. Conclusion: Towards More Sustainable and Resilient Food Systems

In the early hours of 24 February 2022, Russia started a full-scale military invasion of Ukraine that resulted in the deaths and injuries of civilians, as well as the destruction of key infrastructures. The war has also affected global trade, production, and consumption patterns, keeping commodity prices high until 2024 and threatening global food security. Analyzing the long-term and large-scale effects on global food security of conflict-related disruptions in the global food and fertilizer markets is crucial for understanding the overall impact on food security. The paper highlighted that the war resulted in immediate and far-reaching cascading consequences on global food security. Firstly, the war has both short- and long-term implications on Ukraine’s ability to export agricultural products. Secondly, the war prevented farmers from working their fields, and conscription and population displacement caused labor shortages. This situation was exacerbated by limited access to crucial agricultural supplies, such as fertilizers. The war might disrupt the ongoing spring planting campaign and the approaching winter crop harvesting. The war has, as well, some indirect and cascading consequences. Firstly, fertilizer prices are reaching near-record highs, which may severely limit usage and decrease yields. Secondly, as seen during the 2007–2008 food crisis, many countries applied export restrictions, pushing up international market prices and worsening the crisis. Thirdly, panic buying at both country and individual levels is another cascading affect of the war. Finally, the war will delay the sustainable transformation of food systems in many countries. The war may affect the progress toward the SDGs, especially SDG 1 (No Poverty), SDG 2 (Zero hunger), and SDG 12 (Responsible consumption and production).

As the war continues, the potential scope of physical and economic disruptions to food and energy systems raises [73]. Indeed, with the war doubtful to be resolved shortly, its influence on global resource markets will grow, as will the possibility of highly significant ‘ripple effects’ or ‘risk cascades’ on economies and society across the globe. These effects may have swift and severe consequences in regions and industries far from the initial occurrence [73].

Unlike the previous global food-price crisis, driven by the 2007–2008 financial crash, the current upheaval comes after governments and households spent two years coping with the COVID-19 pandemic—the most significant economic shock since World War II. Consequently, humanitarian needs are unprecedented, with climate shocks, conflicts, COVID-19, and growing prices pushing millions closer to famine [78]. The war threatens to worsen the situation by pushing up food prices even further, causing shortages, and creating a global food crisis, especially in conflict-affected countries, such as Yemen, Sudan, Nigeria, and Ethiopia. Meanwhile, from an economic and food security perspective, some countries and regions, including several Middle Eastern, Northern and Sub-Saharan African, and South Asian countries, are more vulnerable than others [10]. Indeed, over 30 countries rely on Russia and Ukraine for at least 30% of their wheat imports, while at least 20 countries rely on those two countries for more than 50% of their wheat imports, making them very sensitive to price shocks and/or supply shortages [46].

However, despite the negative adverse consequences of the war, some opportunities may arise and lessen these consequences. Indeed, early indications show that increased soybean and other oilseed prices will encourage farmers to boost oilseed plantings in the United States and the European Union. Increased wheat acreage in Canada might help cover a part of the global supply gap [32]. Additionally, high fertilizer prices will motivate fertilizer makers to boost production. Likewise, global fertilizer merchants will adjust/restructure trade patterns to transfer the additional supply to the most crucial demand locations (mainly Latin America) [71].

However, for many experts, the failure to reform food systems has allowed the war in Ukraine to spark a third global food price crisis in 15 years. In fact, the consequences of the war on food security are being exacerbated by a variety of underlying rigidities, vulnerabilities, and inefficiencies in global food systems, such as food import dependence, inefficient and speculative grain markets, vicious cycles of conflicts, climate change, poverty, and food insecurity [46]. Consequently, specific urgent actions are required, such as lifting trade obstacles that impede nutrition access, maintaining or expanding social protection programs, safeguarding national nutrition budgets, and mobilizing greater resources for humanitarian aid [16]. Ultimately, governments, philanthropic organizations, the corporate sector, and civil society organizations must all work together to achieve a more resilient and sustainable food system that allows people to eat healthy food reasonably [16]. To prevent recurring global food crises, the international community must address the structural causes of hunger and malnutrition, as well as war, armed conflicts, and pervasive violence [79].

In a world of growing complexity and uncertainty, food systems are under increasing pressure to produce sufficient food for the global population, decrease the environmental impacts of production, and buffer against complex global change [80]. The war is both a warning about the operation of agri-food systems and a booster for their innovation [81]. Food systems are facing several challenges, and addressing them effectively necessitates the creation of research that crosses disciplines and innovates at their intersections to generate diverse solutions that address these challenges’ social, economic, technical, and policy components [82]. Nonetheless, these and subsequent research will serve as a foundation for organizational and government readiness for future shocks, crises, and pandemics. Indeed, the lessons learned from the global response to the COVID-19 pandemic may be used to confront future shocks and contribute to food system transformation [83,84].

Meanwhile, increasing internal and external risks enhance food systems’ shock vulnerability [85]. These shocks could also threaten food supply security [86,87,88] and disrupt many aspects of food systems, resulting in decreased productivity, market disruption, price volatility, and general system instability, disproportionately affecting the most vulnerable and food-insecure [89]. As highlighted by Hendrickson [90]: “The current economic and social organization of our food system presents social, ecological, and economic risks that threaten the long-term capability of humanity to provide its food needs.” (p. 418). Therefore, it is essential to reduce these vulnerabilities and mitigate them by developing policies, technologies, practices, and partnerships that increase the resilience of food systems [80,88]. Further, policymakers in low- and middle-income countries (LMICs) must avoid imposing export limitations or price controls, which might amplify the rise in commodity prices. With increasing inflation, tighter financial conditions, and high debt levels restricting policy options, funding may be redirected toward targeted aid for vulnerable households [91].

In the context of the Russia–Ukraine war, the transition toward a healthy, equitable, and ecologically sustainable food system must be strengthened, not abandoned [92]. A new CGIAR report outlines seven key initiatives for global policymakers to minimize supply and price shocks and improve food systems’ resilience to future crises [93]:

1. Invest in real-time analyses: real-time monitoring of food and input price volatility, together with country-specific evaluations of food security threats from price shocks and trade restrictions, provides insights into effective global and national policy responses;
2. Scrutinize market interventions: for instance, farmers might get subsidies or lower taxes on goods such as fertilizer and energy;
3. Do not worsen the situation: learning from the 2007–2008 food price crisis, governments should refrain from restricting exports, imposing sanctions that impede food and fertilizer commerce, stockpiling, or panic purchasing, and scrapping environmental initiatives;
4. Target short-term actions: short-term solutions that have proved effective in prior crises and seem relevant now include eliminating biofuel subsidies and mandates, extending social safety nets to the most disadvantaged, and fixing inefficiencies in current subsidies (such as bread subsidies in Egypt and Tunisia);
5. Make long-term investments in climate-friendly agriculture research: knowledge and innovation in agriculture not only contribute significantly to food security and sustainability, but they are also critical to attaining the several SDG 2 goals of eliminating hunger, achieving food security and improving nutrition, and promoting sustainable agriculture [94];
6. Capitalize on promising innovations and scale them up: satellite and remote sensing photos and data may help farmers optimize their use of inputs such as fertilizer, and scientists monitor the spread of pests and diseases; and
7. Invest in policy research: countries, multilateral organizations, and donors must dedicate resources not just to agricultural science but also to research on the optimal policies, programs, and interventions that might increase resilience.

Furthermore, we advocate for debt relief for poor and food-insecure nations, implementing policies that promote food sovereignty and lessen reliance and dependency on a small number of grain crops and exporting countries, and substituting traditional and locally adapted crops for wheat and maize. Finally, funding for agroecology, which relies less on external inputs (e.g., fertilizers, and pesticides) and, instead, valorizes local and endogenous knowledge and resources, as well as agroecological research, should be increased in order to make domestic food systems more resilient to future shocks and crises.

## Data Availability

Data is contained within the article.
